# A 1-year review of anti-Ro/La autoantibody testing in an obstetric population

**DOI:** 10.1007/s11845-025-03935-2

**Published:** 2025-03-21

**Authors:** Elizabeth Tunney, Clare M. Crowley, Claire M. McCarthy, Etaoin Kent

**Affiliations:** Royal College of Surgeons in Ireland, Rotunda Hospital, Parnell Square, Dublin 1, Ireland

**Keywords:** Anti-Ro/La autoantibodies, Congenital heart block, Connective tissue disease, Maternal medicine

## Abstract

**Objectives:**

To evaluate current anti-Ro and anti-La autoantibody ordering patterns, clinical indications for performing these tests, and potential cost–benefit analysis.

**Methods:**

In this retrospective cohort study, patients who underwent autoantibody testing over 1 year were included. Necessary information was obtained from patient electronic records.

**Results:**

In total 47 patients underwent anti-Ro/La autoantibody testing. Of those tested, 11/47 (2%) had positive results and testing was clinically indicated in 26/47 (55%) patients, indicating minimal cost-benefits. The total rate of the cost prescription was €1644.96. The mean time to process tests was 5 days. In those with positive autoantibodies, two infants were diagnosed with congenital heart block and had pacemakers inserted after delivery.

**Conclusion:**

This study found anti-Ro/La autoantibody tests were appropriately ordered in accordance with clinical guidelines. By identifying patients who were autoantibody negative, economic benefits were gained, in terms of antenatal management including reduced frequency of antenatal visits and fetal heart surveillance.

## Background

The association of maternal anti-Ro/SSA and anti-La/SSB autoantibodies with congenital heart block and neonatal lupus is well known [1]. Transplacental passage of anti-Ro autoantibodies is associated with neonatal lupus and congenital heart block, with both conditions affecting 1–2% of fetuses [2]. Isolated congenital heart block (CHB) usually develops at some point between 16 and 24 weeks of gestation, coincident with the increased transfer of maternal immunoglobulin G (IgG) antibodies into the fetal circulation. Furthermore, higher levels of anti-Ro autoantibodies correlate with more significant cardiac complications [3]. Congenital heart block (CHB) usually develops at some point between 16 and 24 weeks of gestation, coincident with the increased transfer of maternal immunoglobulin G (IgG) antibodies into the fetal circulation [4]. Given the irreversibility of anti-SSA/Ro-mediated congenital heart block (CHB), prevention with hydroxychloroquine (a Toll-like receptor antagonist reducing inflammation) can be considered from ten weeks gestation [5]. Contraindications to prescription of hydroxychloroquine include known hypersensitivity to 4-aminoquinoline compounds, e.g., chloroquine, and pre-existing maculopathy of the eye. Given the potential adverse neonatal outcomes, guidelines recommend screening for these autoantibodies preconception or in early pregnancy in women with connective tissue disorders such as systemic lupus erythematosus (SLE), SLE-like disorders, Sjogren’s syndrome and rheumatoid arthritis [4, 6].

To date, data on the testing patterns of these autoantibodies in pregnant women are sparse. Numerous studies evaluating anti-nuclear (ANA) antibody ordering patterns in non-pregnant rheumatological patients have demonstrated that a large proportion of tests are ordered unnecessarily contributing to excessive healthcare expenditure [6–8]. Given that laboratory testing represents a significant workload in medicine, it is an important target for improving healthcare efficiency [9]. Although anti-La/Ro autoantibodies are more specific, and less frequently ordered, than ANA antibodies [10], the presence or absence of these autoantibodies are important in antenatal counselling, as well as ongoing obstetric management. Women identified with these autoantibodies should be treated with hydroxychloroquine before and during pregnancy to reduce the risk of congenital heart block, particularly in women who have had a previous infant affected by congenital heart block [5, 6]. Treatment is also necessary due to the high rate of recurrence associated with congenital heart block, which has been reported to be as high as 12% [11].

Additionally, they require serial fetal heart surveillance, necessitating an increased number of antenatal visits, significantly increasing the economic cost of managing their pregnancies [3, 5]. This is the first study to evaluate anti-Ro/La autoantibody ordering trends in an obstetric population, evaluating specifically the indication for performing these tests. The objectives of this study are to establish current anti-Ro and anti-La autoantibody ordering patterns and assess the clinical indications for performing these tests. Secondly, determine the number of unnecessary tests performed as well as potential cost–benefit analysis. Lastly, evaluate both maternal and neonatal outcomes, comparing those who had the test correctly performed with those who underwent unnecessary testing.

## Methods

This retrospective study was conducted in a tertiary maternity hospital in the Republic of Ireland, with more than 10,000 maternities per year. In our unit, a subset of pregnant women with complex medical needs are reviewed at a weekly consultant-led maternal medicine clinic. In 2021, 304 women attended this clinic and 54 (17.7%) of these had a diagnosis of a rheumatological disorder.

Patients who underwent anti Ro/La autoantibody screening during their pregnancy from January 1, 2021 to December 31, 2021, irrespective of the indication for screening, were included in this study. Indications for performing anti-Ro/La autoantibody testing were completed in accordance with international guidelines [3, 4]. Connective tissue disease screening tests are processed at an external laboratory. The cost to process a single extractable nuclear antigen (ENA) screen is €34.27. Patients eligible for inclusion were obtained from laboratory databases, and necessary information was obtained from patient electronic records.

Data on autoantibody test results, time to process tests, and indication for completing these tests were collected. Demographic data included: patient age, medical condition, hydroxychloroquine consumption, attendance at maternal medicine clinic and neonatal outcome. This data was anonymized and stored in a password encrypted computer. Data was analyzed using Microsoft Excel. Ethical approval was obtained from the local ethical approval committee (REF no 22/010).

## Results

In total 47 women had anti-Ro/La autoantibody screening during the study period. Table 1 summarizes patient demographics, showing a mean maternal age of 34.5 years (range 20–45 years). Of those tested for antibodies, 11/47 (23%) patients had a positive result. The average time to process these tests was 5.8 days (range 2–14 days). Women diagnosed with rheumatoid arthritis [*n* = 16/47 (34%)] most commonly underwent testing. Most patients [39/47 (83%)] attended the maternal medicine clinic. The number of live births was 37/47 (79%), 8/47 (17%) patients have not yet delivered, and 2/47 (4%) patients had a miscarriage.

**Table 1:** Patient demographics

Total Number of patients (*N*=47)	
Age (Years)	
Mean	34.5
Range	20 – 45
Anti-Ro antibodies	
Positive	5
Negative	36
Anti-La antibodies	
Positive	0
Negative	41
Anti-Ro & Anti-La positive	6
Test time return (days)	
Mean	5.8
Range	2 – 14
Not recorded	7 (all antibody negative)
Medical Condition	
RA	17
Sjogren’s	2
SLE	8
MCTD	5
Other	15
Attended Mat Med clinic	
Yes	39
No	8

In those testing positive for anti-Ro/La antibodies [*n* = 11/47 (23%)], 8/11 (73%) patients had known positive anti-Ro/La autoantibodies preconception and 3/8 (38%) of these patients had commenced hydroxychloroquine before conceiving (see Table 2). Hydroxychloroquine was commenced in 2/11 (18%) patients before receiving these results. One patient started hydroxychloroquine at 17 weeks’ gestation. Hydroxychloroquine was not commenced in two patients, the rationale for which is not documented in the healthcare records. There were two infants diagnosed with congenital heart block from this cohort of eleven patients. One lady did not have a known connective tissue disorder and congenital heart block was diagnosed at 21 weeks’ gestation, with subsequent maternal antibodies found to be positive. The second patient had a diagnosis of SLE and had known anti-Ro/La autoantibodies; she was not prescribed hydroxychloroquine. Complete congenital heart block was diagnosed at 23 weeks gestation. Both babies had pacemakers inserted after delivery. The final patient who did not commence hydroxychloroquine had a delay in antibody results until 26 weeks gestation, and it was not commenced at this point. Congenital heart block was not diagnosed in this patient.

**Table 2:** Antibody positive patients (*N* = 11)

	Anti-Ro & Anti-la positive (*N*=6)	Anti-Ro positive (*N*=5)
Age (years)		
Mean	35.8	35.6
Range	24 – 44	29 – 43
Medical condition		
RA	0	1
Sjogren’s	0	1
SLE	1	1
SLE & Sjogren’s	0	1
MCTD	1	0
MCTD & Sjogren’s	1	0
MCTD, SLE & APL	1	0
APL	0	1
Psoriatic Arthritis	1	0
Other	1	0
Attended Mat Med clinic		
Yes	5	5
Test time return (days)		
Mean	5.4	6.6
Range	2 - 14	4 - 9
Commenced HCQ (200mg BD)		
Yes	4	4
-Preconception	2	1
-11–17 weeks	2	3
No	2	1
FH monitoring (every 2wks)		
Yes	5	3
Not documented	1	2
Neonatal outcome		
Live births (denominator)	4	4
- Congenital heart block	2	0
- Normal	2	4
Miscarriage	1	0
Not yet delivered	1	1

Anti-Ro/La autoantibody results were negative in 36/47 (77%) patients. Autoantibody tests were appropriately completed in 26/47 (55%) patients (34.27 per ENA test). The total rate of cost prescription was €1610.69. Identifying patients with negative anti-Ro/La autoantibody screens resulted in reduced antenatal workload, decreasing the financial cost of care for this group.

## Discussion

This study found that, overall, most patients with anti-Ro/La autoantibodies had testing carried out in accordance with the recommended clinical indications. General clinical indications for completing anti-Ro/La autoantibody testing in an obstetric context include all women with a rheumatic musculoskeletal disease, who should as part of baseline investigation either pre-pregnancy or in early pregnancy [11]. There is a large emphasis placed on the importance of preconceptual counselling for these patients to include a detailed summary of previous obstetric history, current disease activity, last flare date and chronic organ damage, and recent serological profile including anti-Ro/La autoantibodies [12]. Despite this, there is often an overuse of antinuclear antibody testing within the hospital environment [13]. However, it is understood that the early and accurate diagnosis of autoimmune diseases in pregnancy can be very challenging even for rheumatology specialists. Undoubtedly, the spectrum of signs and symptoms for connective tissue disorders is extremely wide and often overlaps. Initially, an anti-nuclear antibody related disease must be differentiated from a wide spectrum of disorders, i.e., infections, malignancies, allergic, and adverse drug reactions) presenting with similar signs and symptoms [14].

In our study, we noted another frequent indication to perform anti-Ro/La autoantibody screening was for rheumatoid arthritis [*n* = 17/47 (36%)]. In total 11/47 (23%) patients had positive anti-Ro/La autoantibody results. Hydroxychloroquine was appropriately prescribed and commenced in most of these patients [*n* = 8/11 (73%)]. The identification of patients with negative autoantibody screens resulted in economic benefits in terms of antenatal care and fetal heart surveillance, considering that a single fetal echo costs approximately €300 in addition to postnatal neonatal intensive care unit admission costs to the healthcare institution.

Guidelines state that women with rheumatoid arthritis should be screened for anti-Ro/La autoantibodies [4]; however, these antibodies are less prevalent in this group than in those diagnosed with SLE [15]. Smeele et al. [16] found that women of reproductive age who have rheumatoid factor (RF) positive rheumatoid arthritis were more likely to have positive anti-Ro/La antibodies than those with RF negative rheumatoid arthritis, and perhaps this could be used to stratify testing patterns further. Replication of these findings in future studies may question current guidelines testing recommendations for anti-Ro/La autoantibodies in patients with RF-negative rheumatoid arthritis.

Concurring with international guidelines [4, 5], hydroxychloroquine was commenced in most patients. In those testing positive for anti-Ro autoantibodies at booking, hydroxychloroquine was started at 15-week gestation in two patients, 2 days after confirmed antibody screening, and 14 days before confirmed results in one patient with antiphospholipid syndrome. Commencing hydroxychloroquine ideally preconception or in early pregnancy may reduce the risk of congenital heart block in fetuses exposed to maternal anti-Ro/La autoantibodies, especially in women who had a previous infant diagnosed with congenital heart block [17], reducing recurrence from 21 to 7.5%. Hydroxychloroquine is postulated to inhibit toll-like receptors ligation, limiting inflammation and fibrosis of the atrioventricular node leading to cardiac conduction abnormalities [18].

Given the potential fetal complications of congenital heart block, all patients were appropriately referred and managed through the maternal medicine clinic. Although patients were reviewed routinely at this clinic, it is not clear in 5/11 (45%) cases how often fetal heart rate monitoring was completed. Given the low prevalence (0.7–2%) for congenital heart block in women with no previous affected child [1], it is unclear whether intensive monitoring from 16 to 26 weeks in anti-Ro/La autoantibody positive women is beneficial or cost-effective. Furthermore, there is no proven protocol for prevention and antenatal management of complete congenital heart block [18]. Despite the unproven benefits of congenital heart disease surveillance in this population, our clinical practice is to offer fetal heart auscultation fortnightly from 14- to 26-week gestation.

This study has several strengths and limitations. Evaluation of laboratory data was correlated with indication for testing and clinical outcomes. This provided more insight into the rationale for testing, ensuring anti-Ro/La autoantibodies were ordered in accordance with guideline recommendations. Limitations of this study are the retrospective design, small sample size, and missing data. As a result of this missing data in patients with infants diagnosed with congenital heart block, it is difficult to identify a potential cause for this neonatal outcome. Despite these limitations, this is the first study to evaluate anti-Ro/La autoantibody patterns in an obstetric population, providing a useful insight into current practice to guide future audit and research.

## Conclusion

In conclusion, this study found the majority of anti-Ro/La autoantibody tests were ordered in accordance with recommended clinical indications. Given that these tests were appropriately ordered, economic benefits in terms of antenatal management, including reduced frequency of antenatal visits and fetal heart surveillance in autoantibody-negative patients, were identified. Generally obstetric outcomes were positive, with only two babies diagnosed with congenital heart block. The average time to process tests was five days, allowing efficient prescribing of hydroxychloroquine if required. As this is the first study evaluating anti-Ro/La autoantibody ordering patterns in an obstetric population, it provides an important starting point for future studies to improve patient care in this high-risk obstetric group.

## References

[1] Brito-Zerón P, Izmirly PM, Ramos-Casals M et al (2015) The clinical spectrum of autoimmune congenital heart block. Nat Rev Rheumatol 11(5):301–12. 10.1038/nrrheum.2015.29 10.1038/nrrheum.2015.29 PMC5551504 25800217

[2] Jaeggi E, Laskin C, Hamilton R et al (2010) The importance of the level of maternal anti-Ro/SSA antibodies as a prognostic marker of the development of cardiac neonatal lupus erythematosus: a prospective study of 186 antibody-exposed fetuses and infants. J Am Coll Cardiol 55(24):2778–2784. 10.1016/j.jacc.2010.02.042 20538173 10.1016/j.jacc.2010.02.042

[3] Tunaoglu FS, Yildirim A, Vurali D (2010) Isolated congenital heart block. Tex Heart Inst J 37(5):579–83 PMC2953227 20978575

[4] Sammaritano LR, Bermas BL, Chakravarty EE et al (2020A) 2020 American College of Rheumatology guideline for the management of reproductive health in rheumatic and musculoskeletal diseases. Arthritis Rheumatol 72(4):529–556. 10.1002/art.41191 32090480 10.1002/art.41191

[5] Izmirly P, Kim M, Friedman DM et al (2020) Hydroxychloroquine to prevent recurrent congenital heart block in fetuses of anti-SSA/Ro-positive mothers. J Am Coll Cardiol 76(3):292–302. 10.1016/j.jacc.2020.05.045 32674792 10.1016/j.jacc.2020.05.045 PMC7394202

[6] Andreoli L, Bertsias GK, Agmon-Levin N et al (2017) EULAR recommendations for women’s health and the management of family planning, assisted reproduction, pregnancy and menopause in patients with systemic lupus erythematosus and/or antiphospholipid syndrome. Ann Rheum Dis 76(3):476–485. 10.1136/annrheumdis-2016-209770 10.1136/annrheumdis-2016-209770 PMC5446003 27457513

[7] Man A, Shojania K, Phoon C et al (2013) An evaluation of autoimmune antibody testing patterns in a Canadian health region and an evaluation of a laboratory algorithm aimed at reducing unnecessary testing. Clin Rheumatol 32(5):601–608. 10.1007/s10067-012-2141-y 23292519 10.1007/s10067-012-2141-y

[8] Ahrari A, Barrett SS, Basharat P et al (2020) Appropriateness of laboratory tests in the diagnosis of inflammatory rheumatic diseases among patients newly referred to rheumatologists. Joint Bone Spine 87(6):588–595. 10.1016/j.jbspin.2020.05.007 32522598 10.1016/j.jbspin.2020.05.007

[9] Tan EM, Feltkamp TE, Smolen JS et al (1997) Range of antinuclear antibodies in “healthy” individuals. Arthritis Rheum 40(9):1601–1611. 10.1002/art.1780400909 9324014 10.1002/art.1780400909

[10] Meisgen S, Tingström J, Skog Andreasson A (2017) Environmental and lifestyle factors influencing risk of congenital heart block during pregnancy in anti-Ro/SSA-positive women. RMD Open 3(2):e000520. 10.1136/rmdopen-2017-000520 28955500 10.1136/rmdopen-2017-000520 PMC5604703

[11] Gorman Á, Moore L, O’Brien C et al (2023) Management of rheumatic diseases in the preconception, antenatal and postnatal periods. National Women and Infants Health Programme and The Institute of Obstetricians and Gynaecologists.

[12] Nalli C, Manfredi L, Fredi M et al (2022) Managing puerperium in patients with systemic autoimmune diseases: an update. Expert Rev Clin Immunol 18(4):391–399. 10.1080/1744666X.2022.2050216) 35255770 10.1080/1744666X.2022.2050216

[13] Pri-Paz Basson Y, Neumark E, Kivity S, Tayer-Shifman OE (2024) Mitigating overuse of antinuclear antibody (ANA) testing through educational intervention: a study in internal medicine and neurology departments. Clin Rheumatol 43(12):3935–3939. 10.1007/s10067-024-07180-3 10.1007/s10067-024-07180-3 PMC11582184 39412711

[14] Mahler M, Meroni PL, Bossuyt X, Fritzler MJ (2014) Current concepts and future directions for the assessment of autoantibodies to cellular antigens referred to as anti-nuclear antibodies. J Immunol Res 315179. 10.1155/2014/315179 10.1155/2014/315179 PMC4020446 24868563

[15] Steiner G, Smolen J (2002) Autoantibodies in rheumatoid arthritis and their clinical significance. Arthritis Res 4:S1–5. 10.1186/ar551 10.1186/ar551 PMC3238219 12110150

[16] Smeele HTW, Schreurs MWJ, Costedoat-Chalumeau N, Cornette JMJ, Dolhain RJEM (2021) Low prevalence of anti-SSA (anti-Ro) and anti-SSB (anti-La) autoantibodies in female patients with rheumatoid arthritis with a wish to conceive. RMD Open 7(2):e001727. 10.1136/rmdopen-2021-001727 34244382 10.1136/rmdopen-2021-001727 PMC8268898

[17] Izmirly PM, Costedoat-Chalumeau N, Pisoni CN et al (2012) Maternal use of hydroxychloroquine is associated with a reduced risk of recurrent anti-SSA/Ro-antibody-associated cardiac manifestations of neonatal lupus. Circulation. 3;126(1):76–82. 10.1161/CIRCULATIONAHA.111.089268 10.1161/CIRCULATIONAHA.111.089268 PMC3437628 22626746

[18] Saxena A, Izmirly PM, Mendez B, Buyon JP, Friedman DM (2014) Prevention and treatment in utero of autoimmune-associated congenital heart block. Cardiol Rev 22(6):263–7. 10.1097/CRD.0000000000000026 10.1097/CRD.0000000000000026 PMC4539276 25050975

